# Recent Advances in Health Impact Assessment and Health in All Policies Implementation: Lessons from an International Convening in Barcelona

**DOI:** 10.3390/ijerph17217714

**Published:** 2020-10-22

**Authors:** Bethany Rogerson, Ruth Lindberg, Fran Baum, Carlos Dora, Fiona Haigh, Arielle McInnis Simoncelli, Lee Parry Williams, Genandrialine Peralta, Keshia M. Pollack Porter, Orielle Solar

**Affiliations:** 1 Health Impact Project, Washington, DC 20004, USA; brogerson@pewtrusts.org; 2 Southgate Institute for Health, Society and Equity, WHO Collaborating Centre on the Social, Political and Commercial Determinants of Health Equity at Flinders University, Adelaide, SA 5042, Australia; fran.baum@flinders.edu.au; 3 Mailman School of Public Health, Columbia University, New York, NY 10032, USA; carlos.f.dora@gmail.com; 4 Health Equity Research Development Unit, Sydney Local Health District, University of New South Wales, Sydney 2033, Australia; f.haigh@unsw.edu.au; 5 Arielle Simoncelli Consulting, Harrisburg, PA 17103, USA; arielle.simoncelli@gmail.com; 6 Wales Health Impact Assessment Support Unit, Policy and International Health, World Health Organization Collaborating Centre on Investment for Health & Well-being, Public Health Wales, Mold CH7 1PZ, Wales, UK; Lee.ParryWilliams@wales.nhs.uk; 7 Asian Development Bank, Mandaluyong City 1550, Philippines; glperalta99@gmail.com; 8 Department of Health Policy and Management, Johns Hopkins Bloomberg School of Public Health, Baltimore, MD 21205, USA; kpollac1@jhu.edu; 9 Work, Employment, Equity and Health Program, Latin American Social Sciences Faculty (FLACSO), Santiago 7630412, Chile; orielle.solar@flacsochile.org; 10 Chile and Public Health Institute, Faculty of Medicine, University of Chile, Santiago 8380000, Chile

**Keywords:** health impact assessment, health in all policies, equity, health policy, cross-sector collaboration

## Abstract

Health Impact Assessment (HIA) and Health in All Policies (HiAP) are policy tools used to include health considerations in decision-making processes across sectors such as transportation, education, and criminal justice that can play a role in improving health and equity. This article summarizes proceedings from an international convening of HIA and HiAP experts held in July 2019 in Barcelona, Spain. The presentations and panel discussions included different models, best practices, and lessons learned, including from government, international banks, think tanks, and academia. Participants discussed ideas from around the world for cross-sector collaboration to advance health. The convening covered the following topics: community engagement, building greater understanding of and support for HiAP, and exploring how mandates for HIA and HiAP approaches may advance health and equity.

## 1. Introduction

Research shows that social, physical, and environmental conditions play a significant role in determining the length and quality of people’s lives [1,2]. Decisions made in sectors such as housing, transportation, education, and criminal justice have important consequences for health. For example, transportation policies can shape factors such as traffic safety, air pollution, and how people can access goods, services, employment, and social networks—factors which in turn affect rates of injury, asthma, and stress among the population [3,4,5,6].

Health in All Policies (HiAP) is a framework that uses a range of tools on an ongoing basis to examine and address the nuanced and complex connections between decisions in a range of sectors and health outcomes. The World Health Organization (WHO) defines HiAP as “an approach to public policies across sectors that systematically takes into account the health implications of decisions, seeks synergies, and avoids harmful health impacts in order to improve population health and health equity” [7,8]. HiAP is based on the notion of understanding the potential health impacts of policy decisions made in various sectors, and then working with sectors such as housing, transportation, energy, and education to address potential harms to health and maximize health benefits. HiAP uses a variety of tools, including health impact assessment (HIA) and health lens analysis [9], both to determine the expected health impact of proposed actions and to establish a basis for working across sectors. The Public Health Institute lists cross-sector collaboration, health equity, and stakeholder engagement among the key elements of HiAP [10,11]. HIA is one of several strategies that can be used to analyze the potential health effects of discrete, proposed decisions within the HiAP framework. The Gothenburg consensus paper states that HIA “is a combination of procedures, methods and tools by which a policy, program or project may be judged as to its potential effects on the health of a population, and the distribution of those effects within the population” [12]. HIAs can be completed over a few weeks or months using a “rapid” or “desktop” model, or take longer, using an “intermediate” approach using available data or a “comprehensive” approach involving primary data collection, both of which can take several months to more than a year to complete.

Applications and practice of HiAP and HIA can vary significantly across different geographic, organizational, jurisdictional, and cultural contexts. For example, most projects in the United States are not mandated, but are conducted voluntarily by government agencies and/or nonprofit organizations, and are supported by philanthropy [13]. In countries including Wales, Thailand, and others, HIAs are systematically integrated into public decision-making and mandated under certain circumstances [14]. Environmental impact assessments (EIAs) are required for large projects—such as infrastructure, transportation, energy, or mining projects—in most countries worldwide to meet loan and permitting specifications [14]. EIAs sometimes include health considerations, such as impacts related to noise and air quality, and there are examples of HIAs incorporated into or conducted in parallel to EIAs.

While HiAP offers a framework that governments and other organizations can use to advance the aim of creating a healthier, more equitable, and sustainable society, the approach also faces implementation challenges and limitations. For example, the effects of HiAP efforts can be limited by the extent to which the public policies assessed through a HiAP approach can improve health determinants and by broader national, regional, or global policies and factors [15]. Additionally, although addressing health inequities is a core component of HiAP, if these initiatives focus on policies that affect health determinants but not the underlying drivers of health inequities, such as racism or power inequities, the effects on reducing health inequities can be limited [15].

This article summarizes the main themes from expert presentations and panel discussions at an international convening on HiAP and HIA in Barcelona with 40 attendees from 11 countries: Australia, Canada, Chile, Colombia, England, Philippines, Scotland, Spain, Switzerland, United States, and Wales. It highlights opportunities for practitioners and their partners around the globe to strengthen and improve their HiAP and HIA work. By reporting on the issues and ideas discussed at the convening, the authors aim to continue dialogue and strengthen HiAP and HIA practice by further sharing examples and insights with practitioners.

## 2. Convening Overview and Goals

International experts in HiAP and HIA gathered in Barcelona, Spain in July 2019 to discuss incorporating health considerations in policy decisions in multiple sectors, such as housing, transportation, and education. The convening, titled Advancing Health and Health Equity: Lessons from Around the Globe, was hosted by the Health Impact Project, a collaboration of the Robert Wood Johnson Foundation and The Pew Charitable Trusts. The event brought together experts who have developed, used, evaluated, and/or funded well-tested or innovative methods for policy analysis and cross-sector collaboration—including HiAP and HIA—to advance population health and health equity. Sessions, including presentations from around the world, focused on different models, best practices, and lessons learned, as well as emerging ideas for cross-sector collaboration. Attendees represented government, academic, and non-profit organizations. 

The primary goals of the convening were to: (1) strengthen respective work through shared learning; (2) spark new ideas for application in attendees’ home countries; (3) increase understanding of recent research and projects related to HIA/HiAP; and (4) create new and stronger networks among individuals and organizations. Data from the convening evaluation suggest these goals were met. For example, 89% of respondents strongly agreed or agreed that the meeting facilitated networking and dialogue, and 90% strongly agreed or agreed that the meeting provided information that will help inform participants’ future work.

Secondary goals of the convening were to:

1. Complement other organizations and conferences that convene practitioners of HIA and HiAP, such as the Society of Practitioners of Health Impact Assessment’s (SOPHIA) Practitioner Workshop, the annual European Public Health Association meeting, and the International Association for Impact Assessment. The convening aimed to provide additional opportunities for practitioners—defined as individuals who conduct or have conducted HIA and/or HiAP—to share strategies and lessons learned, network, and discuss practice challenges. It built on topics and connections made through previous events to support the HIA/HiAP fields, including the 2013 International HIA conference and the 2015 U.S national HIA meeting.
2. Connect HIA and HiAP practitioners with researchers and policy professionals with expertise in a wide range of areas including criminal justice, education, economics, family and child policy, and housing by taking place directly following the international conference of the Association for Public Policy Analysis and Management (APPAM).

The presenters and titles of presentation and panel discussions are listed in Table 1. Each session is summarized in this article. 

## 3. Health in All Policies: Governing for Health, Equity and Sustainability

**Figure 1:**
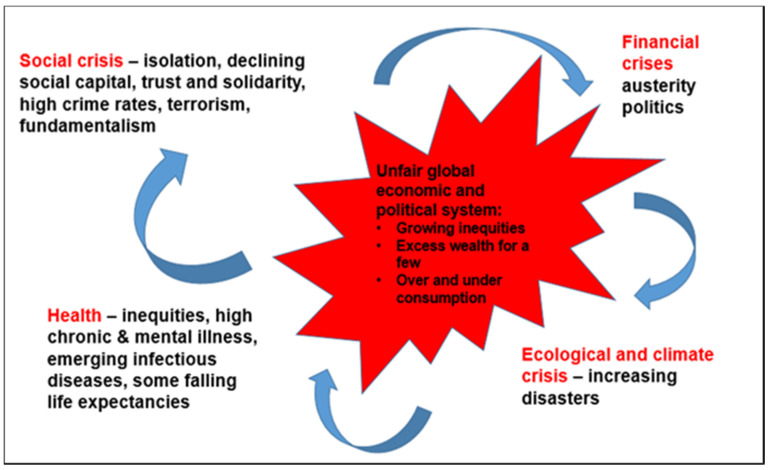
Interrelated systems and crises requiring cross-sector solutions. Source: Baum, F. (2019), *Governing for Health: Advancing Health and Equity through Policy and Advocacy* (New York: Oxford University Press), p. 10.

The opening presentation set the foundation for the day’s discussion with an overview of the challenges and conditions that require cross-sector solutions (Figure 1) [16], and the government infrastructure and capacity that must be put in place to achieve those solutions. 

The challenges are global, stem from a complex array of factors, and encompass the following crises:

- Social: Trust in government and societal institutions is falling;
- Financial: The global financial crisis of 2008 led to austerity measures in many countries and the continued widening of economic inequities;
- Political: Trust in politicians is declining and electoral processes are seen as open to manipulation;
- Ecological: Climate crisis, with its threats to economic well-being and health, is accelerating.

These social, financial, political, and ecological issues are fueling increased health and social inequities. Life expectancies for some populations are decreasing, chronic disease incidence is rising, and loneliness and social distrust are increasing.

Addressing these challenges requires work across various sectors to find innovative, creative, and health-promoting solutions, which the HiAP framework can help identify and advance. In South Australia, for example, the HiAP initiative between government departments aims to find win-win solutions to problems and to establish relationships built upon trust and which can use co-benefit approaches [17]. The evaluation of the project using a narrative theory of change indicated that its varied initiatives did, in all probability, lead to health gains [18].

In one city, for example, the urban planning and health departments identified shared goals related to bicycle infrastructure. The planning department’s goal to create a “vibrant” city converged with the health department’s goal of encouraging cycling for health benefits [18]. The HiAP initiative contributed to ensuring that the 30 year plan for the city contained multiple initiatives designed to promote health including increased walkability, improved public transport, and more livable suburbs [19].

HiAP can help governments take action to create a healthier, more equitable and sustainable society. As a governance mechanism, HiAP relies on some means of working with other sectors to determine how they contribute to or detract from health and well-being. Very commonly this is through procedures such as HIA or, in the South Australian case, a health lens analysis to determine the health impacts of particular sectors. Health lens analysis shares characteristics with HIA; however, it is often used to help inform policy development at a conceptual stage or shape policy priorities [9,20]. These assessments must be followed by a process to determine how to enhance aspects of a project, program, plan or policy that promote health and reduce potential risks and negative health outcomes. This is a political process: advancing a health equity agenda requires governments to consider health and equity across sectors despite competing internal and external pressures and priorities.

## 4. Novel Methods and Approaches to Health-Focused Policy Analysis

The first panel discussion shared innovative methods and approaches used by HIA and HiAP practitioners to conduct health-focused analyses of proposed policies. Factors such as the policymaking timeframe, available budget, and staffing capacity are all important considerations HIA and HiAP practitioners must weigh when determining the best way to incorporate public health data and knowledge as well as stakeholder engagement into a decision-making process. Two innovative tools were emphasized during this session. One is a health note, which is a brief, nonpartisan summary based on the best available research to help policymakers learn about the potential health impacts of proposed legislation and policies [21]. Health notes advance HiAP by helping legislators understand how policies on education, housing and employment can affect health and well-being. While health notes incorporate aspects of the HIA process, they can be completed more rapidly than an HIA, they do not include recommendations, and stakeholders are primarily legislators and their staff and expert peer reviewers. The Pew Charitable Trusts is piloting and evaluating health notes in several jurisdictions in the United States. As of August 2020, Pew has directly completed nine notes in two states (Colorado, Indiana) and provided training and technical assistance to three independent, nonprofit research and policy organizations in Ohio, California, and North Carolina through a grant to the Center on Budget and Policy Priorities to expand the health note pilot program to those states, resulting in six additional health notes. The notes have examined the potential health and equity implications of bills that would create a youth workforce readiness program, prohibit charging fees for all-day public kindergarten, and make it easier for homeless youth to obtain state-issued identification such as a birth certificate or driver’s license, among other topics [21].

The second tool adapted HIA approaches to fit with fast-paced and increasingly complex policy environments and decision-making timelines. The Centre for Primary Health Care and Equity, which is based in Sydney, uses a multi-level approach to institutionalize HIA. Through projects to understand how health and health equity can fit into the organizational environment, the Centre identifies barriers, facilitators, and points of entry for health equity-focused thinking, developing high level organizational drivers and tailored tools and frameworks to address health in different decision-making contexts.

Traditionally HIAs have been carried out on a proposal in development; however, an HIA on a draft proposal often comes too late to have significant influence. In addition, because major proposals can be multistage and multiyear, conducting an HIA at one point in time can be inadequate, especially if the assessment is not integrated into the decision-making process. The Centre is now completing HIAs as part of the proposal development stage and over multiple stages of a policy’s lifecycle. At times, when a comprehensive HIA is not necessary or possible given time constraints, the Centre applies methods that may be part of an HIA process. For example, the Centre may engage stakeholders regarding data findings to develop an understanding of the context and complexities, and identify points of intervention.

Several key themes emerged from the panel discussion. While adaptations of HIAs can help meet time and resource constraints, there are trade-offs and risks. Shorter time frames may be used as a rationale for not engaging stakeholders in the communities whose health and well-being will be affected by decision-making. Limiting analysis to readily available data or to elements that can be quantified risks diminishing the value HIA brings by taking a broad examination of health and elevating a range of data sources, including perspectives of community members that are most likely to be affected by a proposed decision, and by including consideration of structural and systemic determinants of health and equity.

## 5. Using HIA to Frame the International Response to the Prevention of the Health Effects of Air Pollution

The next presentation focused on how the WHO has broadened its approach to air pollution and health. Previously, the focus was on informing about the evidence for health impacts of different levels of air pollutants, in the form of Air Quality Guidelines, which can be used by countries to establish standards (which then need to be enforced). Over the last five years, an additional focus was put on encouraging the sectors that pollute to integrate health into their decisions, using a HiAP approach. The presentation described how this was mainstreamed within WHO programs and into discussions with member states about how to enhance the global response to the health damage caused by air pollution. Air pollution has only recently been recognized as one of the main risk factors for noncommunicable diseases by the WHO and the United Nations General Assembly in 2018, but it causes as many deaths as tobacco. Because air pollution is generated by different sectors, such as transportation and energy production, the public health response requires the engagement of those sectors.

World Health Assembly resolutions using the above framing were adopted by ministers of health in 2015 and 2016 and reflect the shift in how the WHO has approached air pollution and health to include increased focus on cross-sector collaboration and engagement. A first WHO global conference on air pollution and health then involved stakeholders from other sectors, such as energy, urban planning, housing, and from disease programs (Child Health, Non-Communicable Diseases), and was held in 2018 at the WHO headquarters in Geneva, Switzerland, to engage them in implementation. The conference stressed the right to health and to a clean environment, as well as mechanisms for public and stakeholder engagement.

The conference also called for accountability over actions that lead to air pollution and ill health and asked for the “implementation of mechanisms to take stock of actions and progress, and review governance for the prevention of air pollution and related health impacts, and for obtaining additional benefits, including voluntary commitments put forward by the conference.”

Since then, the WHO has continued to use HIA/HiAP as a frame for its subsequent activities related to air pollution and health, including the model for country support it provides to prevent health risks linked to air pollution in cities and in energy policies. The presenter also continues to use HIA/HiAP as the frame for preventing the health effects of air pollution in his role as an advisor with countries, civil society, and philanthropic organizations.

## 6. Statutory and Cross-Sectoral Approaches to Advancing Health and Health Equity

The purpose of this panel was to explore how statutory and cross-sector approaches maintain or advance HIA/HIAP practice, what institutional capacity is needed to establish and support government requirements to conduct HIAs or use a HiAP approach, and the importance of incorporating community engagement and equity-based frames during implementation.

### 6.1. Integrating Health Considerations in the Context of Development in Asia and the Pacific: Applying HIA Requirements and Building HIA Capacity

Development banks and multilateral finance institutions (MFIs) such as the Asian Development Bank (ADB) and the World Bank have safeguard lending policies for infrastructure projects requiring an environmental impact assessment. The ADB health policy is guided by HiAP and recognizes development projects, and infrastructure improvements affecting water, housing, and education, for example, can produce better health outcomes.

Under a technical assistance grant from 2015 to 2018, ADB developed HIA tools and guidelines [22,23,24,25], integrated HIA into selected public health and medical university curricula, conducted training and demonstration projects, and formed the Asia-Pacific HIA Network with active working groups that meet regularly. 

Through the Safeguard Policy Statement (2009), some ADB projects are required to undertake HIA if they are categorized as having potential significant health impacts. Semiannual environmental and social monitoring reports (available on the ADB website) strengthen management of occupational and community health and safety project aspects related to and supportive of social determinants of health. The reports can help identify any noncompliance issues that can in turn be mitigated through corrective actions in a timely manner.

To support HIA practice and capacity building, ADB produced an HIA Framework for Special Economic Zones and industrial parks [26], which provides guidance on maximizing health outcomes while increasing revenues for areas including over 50 million people. Achieving these goals simultaneously can be challenging. The zones or parks are usually located outside of cities and support diverse communities that face many communicable and vector borne diseases. The zone or park infrastructure can support health authorities to create sentinel surveillance, raise awareness for health promotion and prevention, and provide health care facilities. The health records from periodic worker and community member checkups at recognized health care facilities can provide useful data to inform HIA baseline information and other surveillance systems.

### 6.2. Insights on the Evolution of HIA Practice and Laws in Wales

Since 1998 Wales has been going through the process of establishing limited self-government within the United Kingdom. This process is known as devolution and it has enabled the Welsh government to develop policies and pass laws over a range of topics including health, education, and planning. That year, the government published Better Health, Better Wales (1998), which advocated for the use of HIA to address determinants of health and health inequalities within a HiAP approach [27]. Notable policies followed, including the groundbreaking Well-being of Future Generations Act 2015 [28]. It places a duty on public bodies to contribute to achieving national well-being goals, including health and equality.

A major milestone for HIA practice in Wales was when the Welsh Government passed the Public Health Act 2017 [29]. The Act places a duty on the government to make regulations that require all public bodies to undertake HIA in specified circumstances. The Public Health Act mandates that Public Health Wales provide assistance (as yet undefined) to all public bodies within the implementation of the new HIA measure. Three elements have been instrumental in advancing HIA in Wales.

1. A supportive policy environment: In publishing Better Health–Better Wales (1998), the devolved administration was making it explicit that considerations for health and well-being would be central in all policy making in Wales. In addition, they made it clear that HIA was the tool to be used to support HiAP. Without that clear vision it would have made it more difficult to advance the practice of HIA in Wales.
2. Strong advocacy: The Welsh government funded the Unit to advocate for and advance HIA as a way of gaining wider understanding and application of HiAP. The advocacy was driven by research and voluntary HIA application, and building relationships across sectors, organizations, and communities. The Unit provides technical assistance and encourages organizations to take ownership of the HIA rather than build expectation that the Unit will conduct it.
3. Training and capacity building: The Unit’s establishment enabled development of programs, briefings, and materials for different settings and sectors. It developed an HIA methodology to ensure a consistent, value-based approach across Wales. The Unit recently revised their training and capacity framework/strategy [30] to prepare for the anticipated increase in demand from the introduction of statutory HIA.

### 6.3. Bridging National and Local Government and Engaging Community: Reflections from Latin America

Historically, government entities in Latin America and the Caribbean have collaborated across sectors at the local level. Generally, these cross-sector collaborations are driven and sustained by community advocacy. Since the 1970s, some regional governments have decentralized decision-making processes, increasing power at the local level. Citizen engagement in policy making is one of the suggested factors driving cross-sector collaboration.

In this context, the panelist presented the opportunities and challenges that municipalities or local governments face in addressing health and health equity.

Local entities have responsibility for the management of sectors such as education, environment, health, and housing. These jurisdictions can bring together key actors and social organizations to enhance communication and engagement with communities. At the local level, it is possible for interventions and strategies to be driven and implemented top-down by the government or by grassroots organizing efforts.

However, in some countries in these regions, local governments may not have the authority to address underlying systems and structures that may support or hinder community engagement. Additionally, promoting and measuring citizen participation in decision-making at the local level can also be a challenge. The health sector can use metrics to measure the outcomes—not simply the outputs—of engagement efforts. For example, if a longer term goal of engagement is to improve citizen participation in decision-making processes--and in the shorter term, improve people’s understanding of how to participate--it may be more meaningful to measure changes in people’s knowledge of engagement opportunities, rather than the number of ways for citizens to provide input [31].

To overcome challenges in embedding health considerations in a range of sectors, the health sector can identify ways to support other sectors’ agendas and identify mutually beneficial approaches. This could include, for example, health sector efforts and activities to incorporate stakeholders’ varied perspectives, identifying ways that sectors may define and use terms differently, and acknowledging differences in power and resources between individuals and organizations that influence how decisions are made that affect health.

## 7. Health in All Policies Implementation: Lessons from Catalonia

Over the last decade, Europe has made significant efforts to improve health and health equity using HiAP. In Barcelona, for example, cross-sector collaboration and consideration of health contributed to the development of the city’s vast bike lane infrastructure and Superblocks, which are clusters of nine blocks across the city where traffic is restricted to promote urban mobility and accessible public space for pedestrians and cyclists. The goal of this panel was to highlight ways in which HiAP has been implemented in Barcelona and the Catalonian region, the conditions that support HiAP practice, and lessons learned. 

The first panelist highlighted the Interministerial Plan of Public Health (PINSAP) which adheres to the WHO’s HiAP Framework for Country Action and which was unanimously approved by all political parties. The two stages of PINSAP were designed to: (1) improve population health and address the health determinants, and (2) focus on health as a fundamental human right, emphasize the relationship between health and sustainable development within the framework of the 2030 UN Sustainable Development Goals Agenda, and improve cross-sector collaboration.

The second panelist shared how the Catalan Health Service is prioritizing the use of health resources to address social determinants of health and support well-being. For example, the department is prioritizing overcoming systemic barriers to accessing health care, such as stigma and discrimination, with a focus on transgender people’s access to health services, early diagnosis and treatment of endometriosis, and the provision of sexual and reproductive health care in communities.

The third panelist highlighted the 2017–2027 Strategy for Inclusion and Reducing Social Inequality in Barcelona [32]. This strategy sparked new policies and has promoted existing multisector approaches and actions. For example, the Barcelona Public Health Agency is contributing to the development of a mental health plan that is designed to improve mental health care and provide equal opportunities without discrimination. The agency also participates in the Barcelona Circuit against Gender Violence, a coordinated network connecting services—including health care, social, police, judicial, and educational services—to prevent and combat violence against women. 

Panelists underscored three lessons from their experience with implementation of HiAP, which were consistent with reflections and experiences of convening participants from other parts of the world: (1) Engage community members in decision-making about potential and in progress HiAP interventions to build horizontal relationships between the local authority and the general public; (2) Public health professionals must give mutual consideration for the priorities of other sectors and understanding of what actions they can take to address health; and (3) Those with an interest in implementing HiAP approaches should understand that resistance to HiAP is not unusual, but can be overcome.

## 8. Discussion: Overarching Convening Themes

Despite the range of topics, types of HIA/HiAP approaches, and varying geopolitical contexts, several overarching themes resonated throughout the convening.

*Engaging community and increasing transparency for multisectoral solutions:* The health sector alone cannot improve population health and instead requires multisector collaborative efforts to affect key drivers of health that include meaningful community engagement and transparency about goals, research, recommendations, next steps, and outcomes. There are many examples of effective HIA/HiAP initiatives that involve authentic community engagement, which not only illuminates community concerns but also taps into community knowledge about important contextual issues [33]. Engaging communities can also strengthen relationships between government bodies and the constituents they serve by representing their interests in decision-making processes.

*Increasing HiAP awareness, buy-in, support, and capacity:* Multisector collaboration and action also requires broad education, advocacy, and capacity building efforts to inculcate consideration of health in decision-making processes.

Political and organizational will to implement and fund initiatives, as well as the motivation to make changes to improve health based on these efforts, is critical to HiAP success. Challenges to cultivating the will to support and participate in HiAP efforts include lack of strong and clear messages about why other sectors should play a role in improving health and equity. Defining unique roles for all partners—including the private sector, academics, and community-based organizations—will identify specific ways that organizations can contribute or are constrained as they seek to collaborate and can help define and make transparent the varying interests bringing different stakeholders together. Providing evidence about health impacts of a policy or practice may not alone compel organizations to implement recommendations from an HIA/HiAP effort. To address this, HiAP practitioners can identify decision-making drivers in other sectors, and articulate and acknowledge shared or conflicting goals, values, or interests.

Convening participants emphasized the importance of building relationships and engaging stakeholders outside of health in discussions and efforts about building the field and practice of HiAP. Entities such as the WHO play a key role in increasing understanding of support for and training about HiAP [34].

Although awareness is increasing within the public health profession, university courses, continuing education opportunities, and training are critical to advancing HiAP across sectors. Sustaining and expanding practitioner networks, such as the Asia-Pacific HIA Network, SOPHIA, the International Association for Impact Assessment, and the International Union for Health Promotion and Education will also strengthen and advance practice.

HIA/HiAP practitioners also need to make long-term investments in relationship building and partnering to help policymakers see that HIAs should not be viewed as one-off reports or activities, but a broader approach to routinely include health considerations in government decision-making. This would help create champions who see the value of considering health and equity in the policy process.

*Connecting to established goals and ongoing efforts:* To spread and elevate HiAP and to move beyond framing HIA/HiAP as a point-in-time effort, practitioners and leaders can identify connections to existing platforms and infrastructure at multiple levels. At the global level, the United Nations Sustainable Development Goals underpin HiAP efforts and projects and can be used to clarify roles for different organizations and levels of government. As another example, the WHO’s shift to address air pollution and health from a multi-sector perspective aligns with the interdisciplinary and stakeholder engagement principles of HIA practice.

At the organizational level, practitioners and leaders can define how HiAP connects to institutional goals and values and should also identify potential challenges. For example, researchers involved in HIA and HiAP work, such as those in an academic setting, may consider how to transform their work into peer-reviewed manuscripts and describe these efforts as contributions to policy and practice during promotion, tenure, and research grant decisions.

*Selecting from a growing set of HiAP strategies and creating new ones:* As the HIA and HiAP fields continue to grow, practitioners, decision-makers, community residents, and other stakeholders can benefit from the increasing set of strategies available for health-focused policy analysis, such as comprehensive and rapid HIAs, health notes, and health equity lens analyses. Practitioners should also continue to innovate and develop new methods, engaging with a range of disciplines to meet the world’s complex, pressing public health and healthy equity challenges.

Specifically, in determining the appropriate methodology and approach to inform a proposed decision and the lifecycle of a project, panelists offered the following recommendations for practitioners:

- Fully consider the benefits and limitations of the available, potential tools and ensure the selected approach aligns with the core goals of the analysis. For example, a primary goal of ensuring robust, community engagement in a decision-making process may require a different approach than a primary goal of providing policymakers with information during a legislative process about the potential impacts of a proposal on health and health disparities.
- Ensure the selected approach responds to and is appropriate for the geographic, political, and decision-making context.
- Be transparent about any adaptations made to established tools and approaches, and the implications of these adaptations in terms of research quality.

*Considering how HiAP/HIA mandates may advance health and equity:* Mandated approaches present opportunities and challenges. Required application of HiAP/HIA may illuminate health impacts that would otherwise go uncovered or undiscussed. Without funding, awareness, practitioner capacity, and buy-in to implement recommendations resulting from assessments, requirements are likely to yield limited effects on health determinants. As other countries and regions consider institutionalization of these tools and approaches, they should consider ways to potentially accelerate the public health improvements HiAP/HIA aim to achieve.

*Evaluating HiAP and HIA:* To advance HiAP/HIA practice and efficacy, rigorous process and outcome evaluations are needed. There are methodological challenges to assessing complex interventions such as HIA and the role they may play informing policy decisions. Evaluations may be hindered by the difficulty of attributing long-term changes in health outcomes to HiAP, the variety of factors that influence decision-making processes, and a lack of data at the appropriate scale. Measuring changes in the social determinants of health can provide proxy measures and capture whether or how decisions are having the intended effect on health and equity. Building an evaluation evidence base may also help increase buy-in and support for HiAP/HIA incentives, mandates, or funding.

## 9. Conclusions

In response to a strong and growing body of research demonstrating how social, economic, and environmental conditions affect public health and health equity, communities and governments around the globe are turning to HiAP approaches and specific processes like HIA. This convening provided an opportunity to highlight innovative strategies to foster cross-sector collaboration, analyze and inform public policy from a health and equity lens, build capacity for HIA and HiAP work, and engage community residents and organizations in policymaking that can affect their health. The common themes that emerged from the presentations, panels, and convening discussions underscore how HIA and HiAP practitioners can strengthen and advance work in their own jurisdictions. By continuing to share and discuss lessons learned and best practices across countries, HiAP practice can be refined and improved to better address health and equity.

## Figures and Tables

**Table 1:** Overview of Convening Presentations and Panel Discussions.

Presentation or Panel	Presenter and Moderator Names and Affiliations
Health in All Policies: Governing for Health, Equity, and Sustainability (presentation)	Dr. Fran Baum, Southgate Institute for Health, Society and Equity & WHO Collaborating Centre on the Social, Political and Commercial Determinants of Health Equity at Flinders University (presenter)
Novel Methods and Approaches to Health-Focused Policy Analysis (panel discussion)	Dr. Keshia M. Pollack Porter, Johns Hopkins Bloomberg School of Public Health (presenter) Dr. Fiona Haigh, Health Equity Research Development Unit, University of New South Wales (presenter) Ruth Lindberg, Health Impact Project (moderator)
Using Air Pollution as An Entry Point for Health in All Policies Efforts (presentation)	Dr. Carlos Dora, Mailman School of Public Health, Columbia University, formerly with the World Health Organization
Statutory and Cross-Sectoral Approaches to Advancing Health and Health Equity (panel discussion)	Dr. Genandrialine Peralta, University of the Philippines, Diliman (panelist) Lee Parry Williams, Wales Health Impact Assessment Support Unit, Public Health Wales (panelist) Orielle Solar, Latin American Social Sciences Faculty and University of Chile (panelist) Bethany Rogerson, Health Impact Project (moderator)
Health in All Policies Implementation: Lessons from Catalonia (panel discussion)	Dr. Carmen Cabezas, Catalan Department of Health (panelist) Dr. Ramón Escuriet, Catalan Health Service (panelist) Dr. Lucía Artazcoz, Barcelona Public Health Agency (panelist) Arielle McInnis-Simoncelli, former Fulbright fellow at the Johns Hopkins University- Pompeu Fabra University Public Policy Center (moderator)
